# Tenderness-related index and proteolytic enzyme response to the marination of spent hen breast by a protease extracted from *Cordyceps militaris* mushroom

**DOI:** 10.5713/ab.20.0831

**Published:** 2021-04-23

**Authors:** Farouq Heidar Barido, Sung Ki Lee

**Affiliations:** 1 Department of Applied Animal Science, College of Animal Life Sciences, Kangwon National University, Chuncheon 24341, Korea

**Keywords:** *Cordyceps Militaris*, Meat Quality, Proteolytic Enzyme, Spent Hen Meat, Tenderness

## Abstract

**Objective:**

The effects of a crude protease extracted from *Cordyceps militaris* (CM) mushrooms on the postmortem tenderization mechanism and quality improvement in spent hen breast were investigated.

**Methods:**

Different percentages of the crude protease extracted from CM mushrooms were introduced to spent hen breast via spray marination, and its effects on tenderness-related indexes and proteolytic enzymes were compared to papain.

**Results:**

The results indicated that there was a possible improvement by the protease extracted from CM mushroom through the upregulation of endogenous proteolytic enzymes involved in the calpain system, cathepsin-B, and caspase-3 coupled with its nucleotide-specific impact. However, the effect of the protease extracted from CM mushroom was likely dose-dependent, with significant improvements at a minimum level of 4%. Marination with the protease extracted from CM mushroom at this level led to increased protein solubility and an increased myofibrillar fragmentation index. The sarcoplasmic protein and collagen contents seemed to be less affected by the protease extracted from CM mushroom, indicating that substrate hydrolysis was limited to myofibrillar protein. Furthermore the protease extracted from CM mushroom intensified meat product taste due to increasing the inosinic acid content, a highly effective salt that provides umami taste.

**Conclusion:**

The synergistic results of the proteolytic activity and nucleotide-specific effects following treatments suggest that the exogenous protease derived from CM mushroom has the potential for improving the texture of spent hen breast.

## INTRODUCTION

During retail display, meat appearance is the primary factor that affects purchasing intention. However, after processing, meat appearance becomes less influential and is replaced by textural properties that dictate the most important traits for consumer satisfaction [1]. Numerous methods are applied by means of physical and chemical approaches, with physical interventions being less preferable because of their high production costs and negative impact on meat [2]. Consequently, the application of chemical interventions is widely used in the meat processing industry [3]. Nevertheless, with the global consciousness of the importance of a healthy life, shifting to natural-based tenderization is gaining significant interest [4].

In the food industry, mushrooms are utilized as food additives that contribute to flavor variations and are sources of affordable bioactive compounds [5]. Among them, *Cordyceps militaris* (CM) mushroom, a mushroom from the family Clavicepitaceae, exhibits a broad range of functions for health improvements. The metabolites of this mushroom are dominated by cordycepin, adenosine, adenine, polysaccharide and cordyheptapeptide, as well as D-mannitol, which is responsible for its anti-inflammatory, anticancer, immunomodulatory and antifatigue activities [6]. In addition, our previous study suggested that the addition of CM mushroom was not only responsible for flavor enrichment but also led to improvements in samgyetang meat tenderness [7].

It has been proposed that the high content of adenosine 5′-monophosphate (AMP) accounts for the increased liberation of actin as well as the dissociation of actomyosin from myofibrillar protein. Moreover, more free actin could ultimately weaken the cross-links between actin and myosin, thus contributing to the tenderness improvement in meat [8]. However, the effect of AMP on actin and myosin cross-links should not be the only reason for its significant impact on meat tenderness; other factors that are assumed to be involved during the postmortem tenderization mechanism must be explored.

Improved meat tenderness is a result of complex reactions and the degradation of proteins that affect the structure of myofibrillar proteins. Studies have suggested that the dissociation of actomyosin [9] as well as the degradation of key proteins, such as troponin-T, nebulin, dystrophin, titin, and desmin, lead to a significant increase in postmortem tenderness [10]. Endogenous proteolytic enzymes trigger and regulate initial protein hydrolysis reactions. Among proteolytic enzymes, calpains have been widely studied and are assumed to dominate as the main factor during postmortem tenderization. However, study in chicken meat had proven that cathepsin-B enzyme, through extended activation could works more stable than that of calpains [11]. In addition, a key protease involved in apoptosis, cysteinyl aspartate specific protease-3 (caspase-3), has also been mentioned to be involved during postmortem tenderization by promoting the fragmentation of myofibrillar protein as well as a key protein involved in hydrolysis [12].

Limited information is available regarding the postmortem tenderization mechanism of the protease extracted from CM mushroom in spent hens, an affordable protein source with limited utilization. It is widely understood that as age increases, this protease promotes the generation of more cross-links between actin and myosin [13] as well as the formation of collagen, which characterizes stiff and tough meat [14]. In addition, compared to broiler meat, the inferior meat color, unexpectedly strong flavor, and stiff texture make the selling of spent hens for daily consumption nearly impossible and result in the consumption of feedstuffs and concentrated stock preparations [15]. However, spent hens are enriched with a high protein content and omega-3 fatty acids as well as functional peptides. The angiotensin-converting enzyme inhibitory peptides derived from spent hens have been proven to induce the activity of interleukin 10, which is involved in anti-inflammation; inhibit the expression of proinflammatory interleukin 6, and stimulate the activity of dendritic cells and macrophages [16]. Adding value to spent hens by improving their texture properties will not only provide a good affordable source of protein but will also provide economic benefits to the poultry industry. Therefore, this study aims to investigate the effects of the protease extracted from CM mushroom on the postmortem tenderization mechanism as well as quality improvement in spent hen meat.

## MATERIALS AND METHODS

### Preparation of protease powder extracted from *Cordyceps militaris* mushroom

Fresh CM mushrooms were obtained from a local market (Mushtech Co., Ltd., Hoengseong, Korea). The CM mushrooms containing metalloproteases and serine proteases were prepared according to the method proposed by Shin et al [3] with slight modifications. One hundred grams of dry fresh mushroom was ground into small pieces for ease extraction. Well-ground mushrooms were subjected to extraction with an 85% (v/v) ethanolic solution at 4°C±2°C for 24 h. Extracted mixtures without precipitate were subsequently centrifuged at 2,000×g and 4°C for 20 min. After filtration, the filtered solutions were considered to contain the protease extracted from CM mushroom and were subsequently placed in freeze dryers to produce a powder. The freeze-dried powder was stored at −20°C until analysis. A marinade solution containing mushroom powder was prepared by mixing a predetermined concentration of the powder with distilled water followed by an overnight incubation at 2°C±2°C.

### Sample preparation

Sixty-six skinless pectoralis major muscle derived from spent hen breast (72 weeks old) was purchased from a local slaughterhouse (Jung Woo Food Co., Ltd., Gwangju, Korea). All the sample preparation processes were performed in a chilling room at a temperature of 4°C±2°C. To generate similar effects on the breast meat, fat was removed from the breast meat, which was cut into a similar rectangular size of 2.5×2.5×2 cm. Prepared samples were randomly subjected to spraying marination with ±5 mL of a marinade solution containing 0.2 g/100 mL papain as the positive control and a solution containing the protease extracted from CM mushroom at concentrations of 2% (2 g/100 mL), 4% (4 g/100 mL) and 6% (6 g/100 mL) as the treatments. The negative control did not receive any treatment. The marinated samples were enclosed in low-density polyethylene and stored at 2°C±2°C. The proteolytic enzyme activity was determined on days 0, 1, and 4. The data for day 0 were used as a control without any treatment.

### Enzyme activities

Enzyme activity was measured as the proteolytic activity and was determined by using casein as the substrate based on the method described by Ebina et al [17]. One unit of caseinolytic activity was defined as the amount of enzyme that caused an increase of 0.1 absorbance units at 280 nm after a 60 min incubation at 35°C (Sanyo Electric Co., Ltd., Osaka, Japan).

### Shear force value

The marinated samples were packed into plastic bags and subjected to boiling in a water bath (Jeio Tech Co., Ltd., Daejeon, Korea) at 75°C for 35 minutes. The shear force values of the samples were measured using a TA-XT2i Plus instrument (Stable Micro Systems, Surrey, UK) with pre-test speed: 2.0 mm/s; test speed: 1.0 mm/s; post-test speed: 10 mm/s. Each sample was evaluated in three replications.

### Adenosine monophosphate and inosine monophosphate

The method for analyzing the 5′-nucleotide contents (adenosine monophosphate [AMP], inosine monophosphate [IMP]) was according to the method described by Jayasena et al [18] with slight modifications. The determination of 5′-nucleotides was conducted via an HPLC (Waters, Milford, MA, USA) set with a 4.6×150 mm C18 HPLC column (Agilent Technologies, Santa Clara, CA, USA) equipped with a diode array detector (DAD) at a wavelength of 254 nm. The 5′-nucleotide concentrations are expressed as mg of compound per 100 g of cooked matter (mg/100 g).

### Myofibrillar fragmentation index

The determination of the myofibrillar fragmentation index (MFI) was performed according to the method described by Culler et al [19] with slight modifications. Each of the marinated samples was prepared in triplicate. To ensure the elimination of visible fat and connective tissue, the sample was minced into a smaller size, and fat was removed. After hydrolysis with a precooled isolating buffer, the absorbance of the sample supernatant was measured at 540 nm by using a UV spectrophotometer (UV-mini 1240 PC, Shimadzu Corp., Kyoto, Japan). The detected optical density was multiplied by 200, and the result was defined as the MFI.

### Protein solubility

Protein solubility was determined according to the procedure described by Joo et al [20]. Sarcoplasmic proteins were extracted from 2 g minced muscle using 20 mL of ice-cold 0.025 M potassium phosphate buffer (pH 7.2). Total protein (sarcoplasmic + myofibrillar) was extracted from 2 g muscle using 40 mL of ice-cold 1.1 M potassium iodide in 0.1 M phosphate buffer (pH 7.2). Eventually, the myofibrillar protein concentration was determined as the difference between total and sarcoplasmic protein solubility and expressed as (mg protein/g muscle tissue).

### Total and insoluble collagen contents

The total and insoluble collagen contents were determined according to the method described by Jayasena et al [18]. Sample hydrolysis was determined based on the method described by Palka and Daun [21]. The determination of the hydroxyproline content in the sample hydrolysate was calculated via comparison with the standard curve; thus, the result of multiplying the hydroxyproline content by 7.25 was determined as the collagen content and is expressed as mg/g. The extraction of insoluble collagen was performed according to the method described by Liu et al [22] with minor modifications.

### Activity of calpains and cathepsin-B

The activity of endogenous enzymes was determined according to the method described by He et al [12] with slight modifications, in which a substrate (Suc-LY-AMC) was used for calpains, while ARR-AFC was used for determination of cathepsin-B enzyme activity. For the control, the supernatant was replaced with ultra-pure water (ddH_2_O). The absorbance value of the mixture was detected at 380 nm/460 nm (excitation/emission) for calpains and 400 nm/505 nm (excitation/emission) for cathepsin-B. The enzyme activity is shown as the relative absorbance value per min, per mg to the control.

### Activity of caspase-3

Caspase-3 activity was determined according to the method described by He et al [12]. A total of 1.0 mM Ac-DEVD-pNA (dissolved in dimethyl sulfoxide) was used as the substrate and incubated with the supernatant at 37°C for 1 h. The absorbance value of the mixture was detected at a wavelength of 405 nm, and caspase-3 enzyme activity is expressed as the relative absorbance value per min, per mg to the control.

### Statistical analysis

The data analyses performed in this study included one-way analysis of variance using R version 3.6.1 (The R-foundation for Statistical Computing, Vienna, Austria), CRAN mirror and library USA (CA 1) equipped with Agricolae to determine the effects of the protease on proteolytic enzyme activity, AMP and IMP contents, total collagen and insoluble collagen. Two-way multivariate analysis of variance was used for the treatments and storage day to determine their effect on the shear force value, MFI, protein solubility, and proteolytic enzyme activities. A significant value of the mean for each group was continually analyzed using Duncan’s multiple range test, with significance defined as a p-value lower than 0.05.

## RESULTS AND DISCUSSION

### Proteolytic enzyme activity

The enzymatic activities of the protease extracted from CM mushroom are shown in Table 1. A solution containing 6% protease extract powder accounted for 3,547.66 unit/mL, which is significantly lower than the activity of papain, which displayed the highest activity of 9,606.07 unit/mL (p<0.001). In addition, the higher the percentage of the CM extract protease powder within the solution, the higher the activity of the proteolytic enzymes (p<0.001). Moreover, the 4% treatment group exhibited a significantly higher enzyme activity than the 2% group. However, higher activity of proteolytic enzymes does not always contribute to an expected results for meat tenderization, in which mushy texture and overtenderization of meat could occured. Papain as an example, which has been extensively studied for having a broad profile of proteolytic enzyme activity, are characterized to capable of hydrolyzing both connective tissue and myofibrillar proteins [23], thus leading to undesirable quality attributes, such as overtenderized meat, bitter taste, and off-odor [24]. Therefore, these results suggest that the application of the exogenous protease derived from CM mushroom has a milder tenderizing effect to ensure minimal side effects during postmortem tenderization [25].

### Shear force value

The shear force value after treatment the protease extracted from CM mushroom is shown in Table 2. Spent hen breast subjected to treatment with papain had the lowest shear force value throughout the storage day, implying its strong tenderization effect among the treatments (p<0.05). The increase in meat tenderness following treatment with the mushroom extract protease was likely dose-dependent, wherein the 6% treatment group had the lowest shear force value, indicating an increase in tenderness compared to the other treatments (p<0.05). Furthermore, the effect of the 2% marinade solution on spent hen breast did not differ from that of the negative control (p>0.05). The storage day was found to significantly affect meat tenderness in all the treatment groups, and the value on the final storage day was significantly lower than that on the initial storage day (p<0.05). The significant increase in tenderness is assumed to be correlated with the protease enzyme extracted from CM mushroom. Metalloprotease and serine protease derived from CM mushroom could bind to specific sites in myofibrillar protein [26] and lead to endogenous enzyme activation that consequently breaks down the myofibrillar structure as well as that of crucial proteins during postmortem tenderization [27]. This result is in line with our previous findings that the addition of CM mushroom at a minimum concentration of 2% to samgyetang improved its meat texture [7].

### Myofibrillar fragmentation index

The degree of fragmentation of myofibrillar protein is considered as an important index to measure the tenderness level of meat [28]. The quantity of fragmented protein reflects the architectural changes and breakdown of crucial proteins, including troponin-t, desmin, vinculin, nebulin, and titin, as a result of hydrolytic reactions triggered by endogenous enzymes [29]. The MFI value of spent hen breast after treatment the protease extracted from CM mushroom is shown in Table 2. After day 1, the degree of the MFI was highest in spent hen breast samples marinated with papain (p<0.05), followed by those marinated with the crude extract protease at 6%, 4%, 2%, and negative control. Increasing percentage of the mushroom crude extract protease within the marinade solution resulted in a significantly higher MFI than the negative control, except for treatment with the 2% marinade (p>0.05). In terms of papain, a pure protease enzyme generated from papaya latex is known to have a broad spectrum of proteolytic activity, especially the ability to hydrolyze both connective tissue and myofibrillar protein [30]. This study utilized purified papain previously cleared from disturbing substances, which thus had a considerably higher MFI than the highest percentage of the protease extracted from CM mushroom. On the other hand, apart from the additional exogenous protease that possibly improves postmortem meat tenderness [29], the high content of AMP within the CM mushroom could also contribute to the increased degradation of myofibrillar protein. The phosphate chain of AMP can dissociate sarcomere protein through an ionic strength mechanism [9]. The ionic strength of AMP plays a significant role in altering the meat surface environment by promoting a free protein-ion bonds, resulting in a better substantial capacity to retain water, thus affecting muscle cell integrity [31]. The results of this study also indicated that the MFI could be another essential index for meat tenderization.

### Adenosine monophosphate and inosine monophosphate

The concentrations of AMP and IMP were quantified in this study to understand their presence in spent hen breast after treatment with the protease powder extracted from CM mushroom. Higher concentrations of the mushroom extract protease within the marinade solution resulted in a significantly higher IMP content among the samples (p<0.05; Table 3). Each two percent increment improved the IMP concentration within the meat samples (p<0.05). Regardless of the concentration, treatment with the mushroom extract protease significantly increased the content of AMP within spent hen breast compared to both the control and papain groups (p<0.05). This finding is in agreement with our previous study that showed that the AMP content within samgyetang was improved following the addition of CM mushroom [7]. AMP is an essential component for RNA and DNA synthesis found in all living organisms. Studies on flavor enhancement have suggested that this nucleotide is positively correlated with the flavor improvement of chicken soup [18] and samgyetang [7]. On the other hand, CM mushroom has a rich content of nucleotides, including AMP [32]. By performing HPLC, we confirmed that the concentration of AMP within CM mushroom in this study was 0.096 (mg/g), the second highest after cordycepin (data not shown). However, after permeation into the muscle environment, AMP was assumed to be cleaved by adenosine deaminase, an enzyme involved in purine metabolism in animals that breaks down adenosine to generate IMP and urea [33]. As a consequence, this study confirmed an increase in IMP with a higher percentage of the mushroom protease. This finding agrees well with a previously published study on duck meat [18]. Inosinic acid provides an umami taste due to its properties as a highly effective salt [33]. Therefore, the addition of CM mushroom to spent hen breast is assumed to provide a richer taste together with a textural improvement.

### Collagen content

The collagen content in spent hen breast was determined after 24 h following treatment with the mushroom protease and papain. As shown in Table 3, regardless of the concentration of the mushroom protease, both the total collagen and insoluble collagen contents remained unchanged (p>0.05). In contrast, treatment with papain resulted in an enormous decrease in collagen content, indicating a strong hydrolytic effect. Although there are limits of papain for muscle permeation, this enzyme has been widely proven to be capable of hydrolyzing protein molecules, including connective tissue and myofibrillar protein [34]. The effect of the protease extracted from CM mushroom on the collagen content was only outperformed by bromelain [29]. Generally, each protease has a specific substrate and optimal conditions to promote tenderization. The lower collagenic activity of the protease extracted from CM mushroom was likely due to the presence of unnecessary protein within the protease powder, unlike the purified papain enzyme, in addition to its ability to hydrolyze only specific sites of proteins apart from collagen. This finding is in line with the protease extract results from actinidin from kiwifruit, asparagus enzyme [1], and cucumis enzyme [35], which were outperformed by papain in terms of collagen hydrolysis.

### Calpains activity

**Figure 1:**
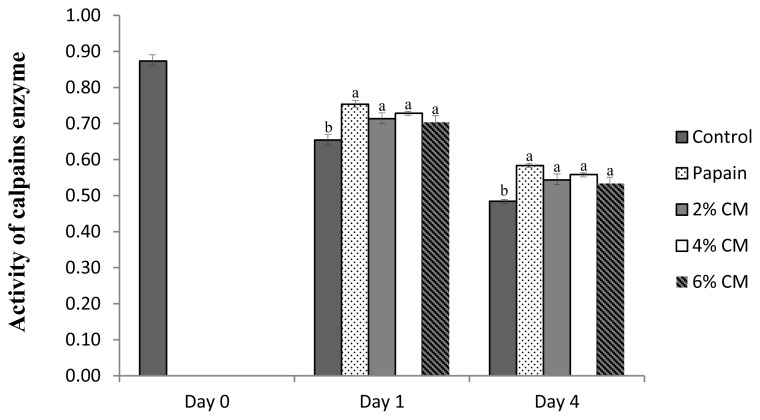
Calpain enzyme activities expressed in (relative absorbance value per min, per mg to control) of spent hen breast after treated with a protease extracted from *Cordyceps militaris* mushroom. Control, breast meat without treatment; 0.2 g/100 mL papain, spent hen breast treated with 0.2 g/100 mL papain; 2% CM, spent hen breast treated with 2% *Cordyceps militaris* mushroom extract protease; 4% CM, spent hen breast treated with 4% *Cordyceps militaris* mushroom extract protease; 6% CM, spent hen breast treated with 6% *Cordyceps militaris* mushroom extract protease. ^a,b^ p<0.05.

Figure 1 displays the effect of the protease extracted from CM mushroom on the activity of calpain in spent hen breast. The activity was found to be the highest on the initial day and significantly decreased with storage time, and the final storage day displayed the lowest activity of calpains in all samples (p<0.05). After day 1, regardless of the concentration of the mushroom extract protease, the treatments maintained a significantly higher activity of calpain activity compared to the negative control (p<0.05) and a similar activity to that of breast meat treated with papain (p>0.05). Moreover, after day 4, although their activity decreased, the calpain enzymes in breast meat treated with 6% and 4% mushroom protease tended to be more stable and likely similar to papain. Postmortem tenderization is thought to result from enzymatic reactions [36], with calpains believed to be a dominant factor in initiating protein hydrolysis [12]. In chicken breast meat, calpains, especially μ-calpain, play a dominant role in inducing the breakdown of key proteins, specifically troponin-t, desmin, and titin, at the early postmortem period during 3 to 12 hours postmortem [37]. However, the mechanism by which the protease extracted from CM mushroom maintains good calpain system activity during storage remains unclear.

### Cathepsin-B enzyme

**Figure 2:**
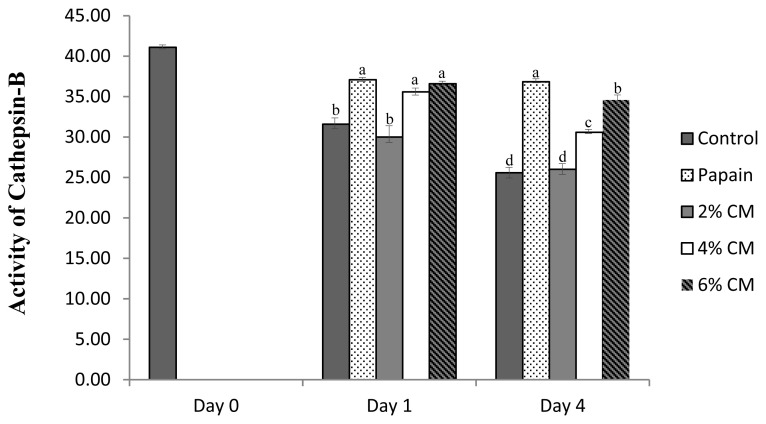
Cathepsin-B enzyme activities expressed in (relative absorbance value per min, per mg to control) of spent hen breast after treated with a protease extracted from *Cordyceps militaris* mushroom. Control, breast meat without treatment; 0.2 g/100 mL papain, spent hen breast treated with 0.2 g/100 mL papain; 2% CM, spent hen breast treated with 2% *Cordyceps militaris* mushroom extract protease; 4% CM, spent hen breast treated with 4% *Cordyceps militaris* mushroom extract protease; 6% CM, spent hen breast treated with 6% *Cordyceps militaris* mushroom extract protease. ^a–d^ p<0.05.

After day 1, the activity of cathepsin-B enzyme in spent hen breast treated with the mushroom extract protease was significantly lower than that in spent hen breast treated with papain (Figure 2). Significant activities of the cathepsin-B enzyme were observed for the groups treated with 6% and 4% marination, with no effect of the 2% marination compared to the negative control. At the final storage day, the cathepsin-B enzyme activity remained highest in papain, followed by the 6%, 4%, and 2% marinades and negative control. During storage, the activity of cathepsin-B was likely stable unless the breast meat samples were not subjected to any treatments. Together with calpains, cathepsins are believed to have a significant function during tenderization. The tenderness-related activity of these enzymes is generated from cathepsins B, D, E, F, H, K, L, and S, with a major contributor still under debated. Although the contribution of cathepsin-B during postmortem tenderization is not clearly explained, along with cathepsin L, these members of the cathepsin family are considered to be more stable after 24 h compared to the well-known cathepsin-D [36]. Many studies believe that this enzyme does not play an important role in increasing meat tenderness postmortem [27]. However, recent findings have revealed that in most fish species, cathepsins B and L initiate tenderization via activation of autophagy, which has been mentioned to be correlated with tenderization, and the calpain system contributes as a secondary enzyme [38]. In addition, a study on the changes in cathepsin activity in beef briskets revealed that cathepsin-B, in particular, may contribute as a stable enzyme to generate improved meat tenderness [11]. Furthermore, this enzyme is thought to be essential after deactivation of calpain activity owing to its characteristics as a more stable enzyme, particularly cathepsin-B and L, which even remain active after 24 hours of heating at 55°C [39].

### Caspase-3 enzyme

**Figure 3:**
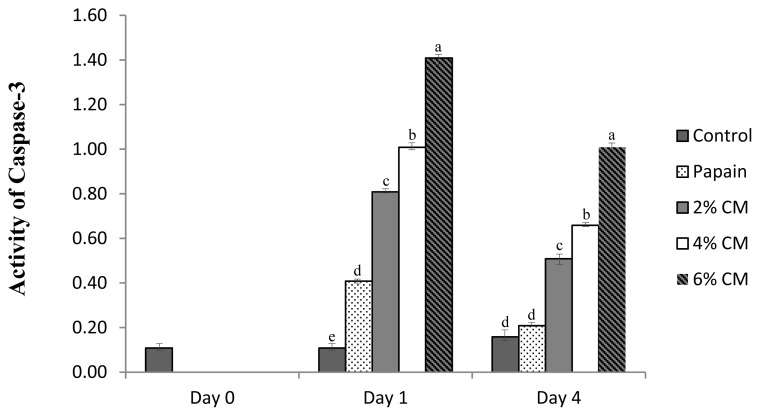
Caspase-3 enzyme activities expressed in (relative absorbance value per min, per mg to control) of spent hen breast after treated with a protease extracted from *Cordyceps militaris* mushroom. Control, breast meat without treatment; 0.2 g/100 mL papain, spent hen breast treated with 0.2 g/100 mL papain; 2% CM, spent hen breast treated with 2% *Cordyceps militaris* mushroom extract protease; 4% CM, spent hen breast treated with 4% *Cordyceps militaris* mushroom extract protease; 6% CM, spent hen breast treated with 6% *Cordyceps militaris* mushroom extract protease. ^a–d^ p<0.05.

As seen in Figure 3, the activity of caspase-3 was upregulated in spent hen breast following the treatments, and the highest percentage of the mushroom protease resulted in the most increased enzyme activity and was significantly higher than that of the papain-treated samples. Regardless of the concentration, the protease extracted from CM mushroom contributed to considerably higher enzyme activity than the negative control. Along with autophagy, apoptosis is considered to be involved in the mechanism of meat tenderization. After an animal is slaughtered, protein fragmentation is initiated, which is generally regulated by the caspase-3 enzyme [40]. In a normal cell, activation of apoptosis occurs to remove harmful compounds and pathogens associated with hypoxia and ischemia [41]. The effect of the caspase-3 enzyme on meat tenderization varies and is likely species-dependent. In chicken meat, after treatment with Ca^2+^, the activity of caspase-3 is upregulated and induces an increase of apoptosis, thus significantly promoting an improvement in meat tenderness [42]. In contrast, a study by He et al [12] on duck breast meat found that although caspase-3 enzyme activity was upregulated by treatment with MDL-28, 170, it did not significantly lead to a remarkable increase in duck meat tenderness. Despite the fact that caspase-3 is believed to play a dominant role during proteolysis, it can be assumed that if it is not a key enzyme, it is at least an essential factor for tenderization of spent layer breast meat. On the other hand, the upregulation of caspase-3 might be attributed to a specific effect of AMP to activate adenosine monophosphate kinase (AMPK), thus inducing apoptosis [43], as well as the bioactive content, namely, cordycepin, to promote a similar activation of AMPK [44].

### Protein solubility

The protein solubility after treatment with the protease extracted from CM mushroom is shown in Table 4. The solubility of myofibrillar protein as well as total protein were significantly affected following treatment with 4% and 6% mushroom protease, as indicated by the higher value than the negative control (p<0.05). The effect of the mushroom protease at 4% and 6% on spent hen breast was not different from that of papain (p>0.05). Regardless of concentration, the insignificant effect of the mushroom protease on the sarcoplasmic protein was observed, while higher solubility was found for the papain group than the negative control. An increase in protein solubility was found along with the storage period in all the enzyme-treated samples (p<0.05). The increased protein solubility following the augmentation of exogenous protease agrees with Naveena et al [35], which found strong solubility effects of cucumis, ginger, and papain in a considered tough buffalo meat. A study by Ha et al [1] reported the ability to extract myofibrillar and sarcoplasmic proteins by papain and bromelain. Therefore, protein solubility is considered an essential factor for tenderness improvement and an accurate indicator of the tenderness level that is strongly correlated with a lower shear force value Shin et al [3]. This result indicates that the possible contribution of CM mushroom to postmortem tenderization was via myofibrillar protein extraction.

## CONCLUSION

The increase in meat tenderness following treatment with the mushroom extract protease was likely dose-dependent, and the mushroom extract protease at a minimum level of 4% within the marinade solution was proven to generate an improved spent hen breast tenderness. The significant contribution to postmortem tenderization by the mushroom extract protease was believed to occur through the upregulation of endogenous proteolytic enzymes, including calpain enzyme, cathepsin-B, and caspase-3, coupled with a nucleotide-specific effect. Thus, these effects lead to an increase in protein extractability as well as fragmentation of myofibrillar protein. Sarcoplasmic protein and collagen content seemed to only be affected by papain, with less effect by the protease extracted from CM mushroom, indicating that substrate hydrolysis was limited to myofibrillar protein. Furthermore, marination with the protease extracted from CM mushroom may also lead to a richer taste of meat products owing to the increased inosinic acid concentration, a highly effective salt that provides umami taste. This study suggests that the crude protease extracted from CM mushroom is a potential enzyme for texture improvement in spent hen breast.

## Figures and Tables

**Table 1:** Proteolytic enzyme activities of a protease extracted from *Cordyceps militaris* mushroom in comparison with papain

Variables	Treatments1)				p-value

	0.2 g/100 mL papain	2% CM protease	4% CM protease	6% CM protease	
Enzyme activity (unit/mL)	9,606.07^a^	990.54^d^	1,921.30^c^	3,547.66^b^	<0.001

1) 0.2 g/100 mL papain, spent hen breast treated with 0.2 g/100 mL papain; 2% CM, spent hen breast treated with 2% *Cordyceps militaris* mushroom extract protease; 4% CM, spent hen breast treated with 4% *Cordyceps militaris* mushroom extract protease; 6% CM, spent hen breast treated with 6% *Cordyceps militaris* mushroom extract protease.

a–d Means within the same row are significantly different among treatment (p<0.05).

**Table 2:** Shear force value (kgf) and myofibrillar fragmentation index of spent hen breast following treatments with a protease extracted from *Cordyceps militaris* mushroom

Storage period (d)	Control	Treatments1)				SEM	p-value		
	
		0.2 g/100 mL papain	2% CM protease	4% CM protease	6% CM protease		Sample	Storage	Sample×storage
Shear force value (kg)									
0	2.79^x^	2.79^x^	2.79^x^	2.79^x^	2.79^x^	0.00	<0.05	<0.05	0.29
1	2.77^a^^x^	2.07^d^^y^	2.75^a^^x^	2.41^b^^y^	2.21^c^^y^	0.13			
4	2.69^a^^x^	1.72^d^^z^	2.52^a^^z^	2.29^b^^y^	1.90^c^^z^	0.18			
Myofibrillar fragmentation index									
0	35.11^x^	35.11^z^	35.11^y^	35.11^x^	35.11^z^	0.06	<0.05	<0.05	0.07
1	34.74^d^^x^	90.71^a^^y^	50.58^c^^x^	74.92^b^^y^	82.43^ab^^y^	10.42			
4	42.65^d^^x^	106.49^a^^x^	59.30^c^^x^	83.11^b^^x^	101.23^a^^x^	12.20			

SEM, standard error of the mean.

1) Control, breast meat without treatment; 0.2 g/100 mL papain, spent hen breast treated with 0.2 g/100 mL papain; 2% CM, spent hen breast treated with 2% *Cordyceps militaris* mushroom extract protease; 4% CM, spent hen breast treated with 4% *Cordyceps militaris* mushroom extract protease; 6% CM, spent hen breast treated with 6% *Cordyceps militaris* mushroom extract protease.

a–d Means within the same row are significantly different among treatment (p<0.05).

x–z Means within the same column are significantly different during storage day (p<0.05).

**Table 3:** AMP, IMP nucleotide content and collagen concentration of spent hen breast following treatments with a protease extracted from *Cordyceps militaris* mushroom.

Variables	Control	Treatments1)				SEM	p-value

		0.2 g/100 mL papain	2% CM protease	4% CM protease	6% CM protease		
AMP (mg/g)	0.34^c^	0.32^c^	0.47^b^	0.58^a^	0.65^a^	0.07	<0.05
IMP (mg/g)	1.36^d^	1.31^d^	2.46^c^	4.59^b^	6.58^a^	1.02	<0.05
Total collagen (mg/g)	2.52^a^	1.07^b^	2.46^a^	2.42^a^	2.39^a^	0.28	<0.05
Insoluble collagen (mg/g)	1.47^a^	0.59^b^	1.52^b^	1.53^b^	1.48^b^	0.14	<0.05

AMP, adenosine monophosphate; IMP, inosine monophosphate; SEM, standard error of the mean.

1) Control, breast meat without treatment; 0.2 g/100 mL papain, spent hen breast treated with 0.2 g/100 mL papain; 2% CM, spent hen breast treated with 2% *Cordyceps militaris* mushroom extract protease; 4% CM, spent hen breast treated with 4% *Cordyceps militaris* mushroom extract protease; 6% CM, spent hen breast treated with 6% *Cordyceps militaris* mushroom extract protease.

a–d Means within the same row are significantly different among treatment (p<0.05).

**Table 4:** Protein solubility of spent hen breast following treatments with a protease extracted from *Cordyceps militaris* mushroom

Storage period (d)	Control	Treatments1)				SEM	p-value		
	
		0.2 g/100 mL papain	2% CM protease	4% CM protease	6% CM protease		Sample	Storage	Sample×storage
Total protein									
0	115.24^z^	115.24^z^	115.24^z^	115.24^z^	115.24^z^	0.03	<0.05	<0.05	0.13
1	121.43^c^^y^	167.8^a^^y^	133.86^b^^y^	153.86^a^^y^	162.99^a^^y^	16.98			
4	139.05^b^^x^	188.42^a^^x^	151.52^b^^x^	171.44^a^^x^	180.61^a^^x^	12.84			
Myofibrillar protein									
0	82.18^z^	82.18^z^	82.18^z^	82.18^z^	82.18^z^	0.03	<0.05	<0.05	0.09
1	85.56^c^^y^	117.97^a^^y^	98.02^b^^y^	113.07^a^^y^	121.39^a^^y^	6.72			
4	96.84^b^^x^	129.25^a^^x^	109.30^b^^x^	124.31^a^^x^	132.67^a^^x^	7.66			
Sarcoplasmic protein									
0	33.06^y^	33.06^z^	33.06^y^	33.06^y^	33.06^y^	0.04	<0.05	<0.05	0.33
1	35.37^xy^	49.83^y^	35.84^xy^	40.79^x^	41.60^x^	5.32			
4	39.21^x^	59.17^x^	42.22^x^	47.13^x^	47.94^x^	7.27			

SEM, standard error of the mean.

1) Control, breast meat without treatment; 0.2g/100mL papain, spent hen breast treated with 0.2g/100mL papain; 2% CM, spent hen breast treated with 2% *Cordyceps militaris* mushroom extract protease; 4% CM, spent hen breast treated with 4% *Cordyceps militaris* mushroom extract protease; 6% CM, spent hen breast treated with 6% *Cordyceps militaris* mushroom extract protease.

a–c Means within the same row are significantly different among treatment (p<0.05).

x–z Means within the same column are significantly different during storage day (p<0.05).
